# Ex-vivo study on the surface quality of corneal lenticule and stroma after low energy femtosecond laser lenticule extraction

**DOI:** 10.1038/s41598-022-13468-8

**Published:** 2022-06-15

**Authors:** Mayank A. Nanavaty, Hasan Naveed, Zahra Ashena, Ritika Mukhija

**Affiliations:** 1 Sussex Eye Hospital, University Hospitals Sussex NHS Foundation Trust, Eastern Road, Brighton, BN2 5BF UK; 2 Brighton and Sussex Medical School, University of Sussex, Falmer, Brighton, BN1 9PX UK; 3 Sussex Eye Laser Clinic, Nuffield Health Brighton Hospital, Warren Road, Woodingdean, Brighton, BN2 6DX UK

**Keywords:** Medical research, Outcomes research

## Abstract

This study aimed to assess the surface quality of cap, stroma and lenticular surfaces created using low-energy femtosecond laser lenticule extraction (Ziemer FEMTO LDV Z8). Twenty-four porcine eyes were divided into four groups (n = 6 each): two with optimal laser power (32%) with posterior curvature equivalent to a spherical correction of -2D and -5D, respectively and the other two with high power (64%) with spherical correction of -2D and -5D respectively. Samples were analysed using scanning electron microscopy (SEM). Surface morphology was evaluated using a standard scoring system; surface relief, surface regularity, extent and position of irregularities were graded by four independent clinicians. Eyes with 32% power and -2D correction had significantly less size of the irregular area than those with -5D; however, no significant difference was found between the two groups with 64% power. When comparing eyes with -2D correction, the size of the irregular area was lesser with 32% power. Surface relief was lesser with -5D correction with 32% power than 64% power. Low-energy femtosecond laser lenticule extraction (Ziemer FEMTO LDV Z8) produces good surface quality results. There is a tendency for smoother surface stromal quality with lower power settings than a higher power.

## Introduction

Femtosecond laser lenticule extraction is an alternative to the laser-assisted in-situ keratomileusis (LASIK) procedure that does not require the excimer laser to perform refractive surgery. The refractive lenticule carved by a femtosecond laser is extracted through a smaller corneal incision. Based on the refraction, the exact shape and thickness of the refractive lenticule are calculated^1^. Previous studies on femtosecond lenticule extraction showed reasonable patient satisfaction^2–6^.

The stroma's surface quality correlates directly with post-operative haze and refraction, which are vital for optimal post-operative vision^7,8^. Previous studies on the surface quality of lenticules using the 200-kHz VisuMax femtosecond laser (Carl Zeiss Meditec, Dublin, CA), with pulse energies between 150 to 195nJ, showed circular holes (dents on the surface) from cavitation bubbles and rough patches^2,7,9^. In another study by Ziabarth et al., circular holes and rough areas were still visible in scanning electron microscopy (SEM) despite using a next-generation VisuMax laser with lower energy^10^. This lenticular extraction with VisuMax is also known as Small Incision Lenticule Extraction (SMILE).

The FEMTO LDV Z8 (Ziemer Ophthalmic Systems AG, Port, Switzerland) is a femtosecond laser with high-frequency and a three-dimensional cut setting. The technical description of this laser system is already published elsewhere^11^. FEMTO LDV Z8 is a versatile laser and can be used for several procedures^12–15^. FEMTO LDV Z8 has been upgraded to provide a new function for creating lenticules^16^. This is named Corneal Lenticule Extraction for Advanced Refractive correction (CLEAR). This handpiece creates focused laser pulses in the low nanojoules range (< 100 nJ) for photodisruption^6^. Thus, the advantage of the low-energy concept is decreased stromal gas generation and an accurate laser focus. Due to its high frequency (several megahertz), it has overlapping spots as the pulses are not at spot distance from each other. The arm of the laser is articulated, moveable and adaptable to position and height. The laser pulses are delivered from the laser source through this arm when docked to the eye's surface. In addition, after the docking, with the FEMTO LDV Z8, it is possible to re-centre the treatment area^6,17^.

While raising the femtosecond laser power increases the chance of a denser opaque bubble layer, reducing the laser power increases the tissue bridges; both can impact the quality of the stroma, cap, lenticule and affect the surgery and outcomes^18,19^. The refractive error in addition to the laser power may influence formation of bubble layer and incidence of tissue bridges. Therefore, we designed this *ex-vivo* study to examine the surface quality of cap, stroma and opposing lenticular surfaces created using Ziemer FEMTO LDV Z8 for − 2.0D (diopter) and − 5.0D myopic spherical refraction with low and high laser power settings. The lower settings correspond to optimal laser power, and the higher power is doubled the optimal power setting.

## Methods

Fresh porcine eyes (n = 24) were procured from the butchers for this study. They were stored in a wet chamber at + 4° C and used within 5 h of retrieval. Before surgery, the eyes were held at room temperature for 2 h to allow deswelling of the corneal stroma. IRB/ethics approvals were not required as the butchers would have discarded the eyes used for this study.

**Figure 1:**
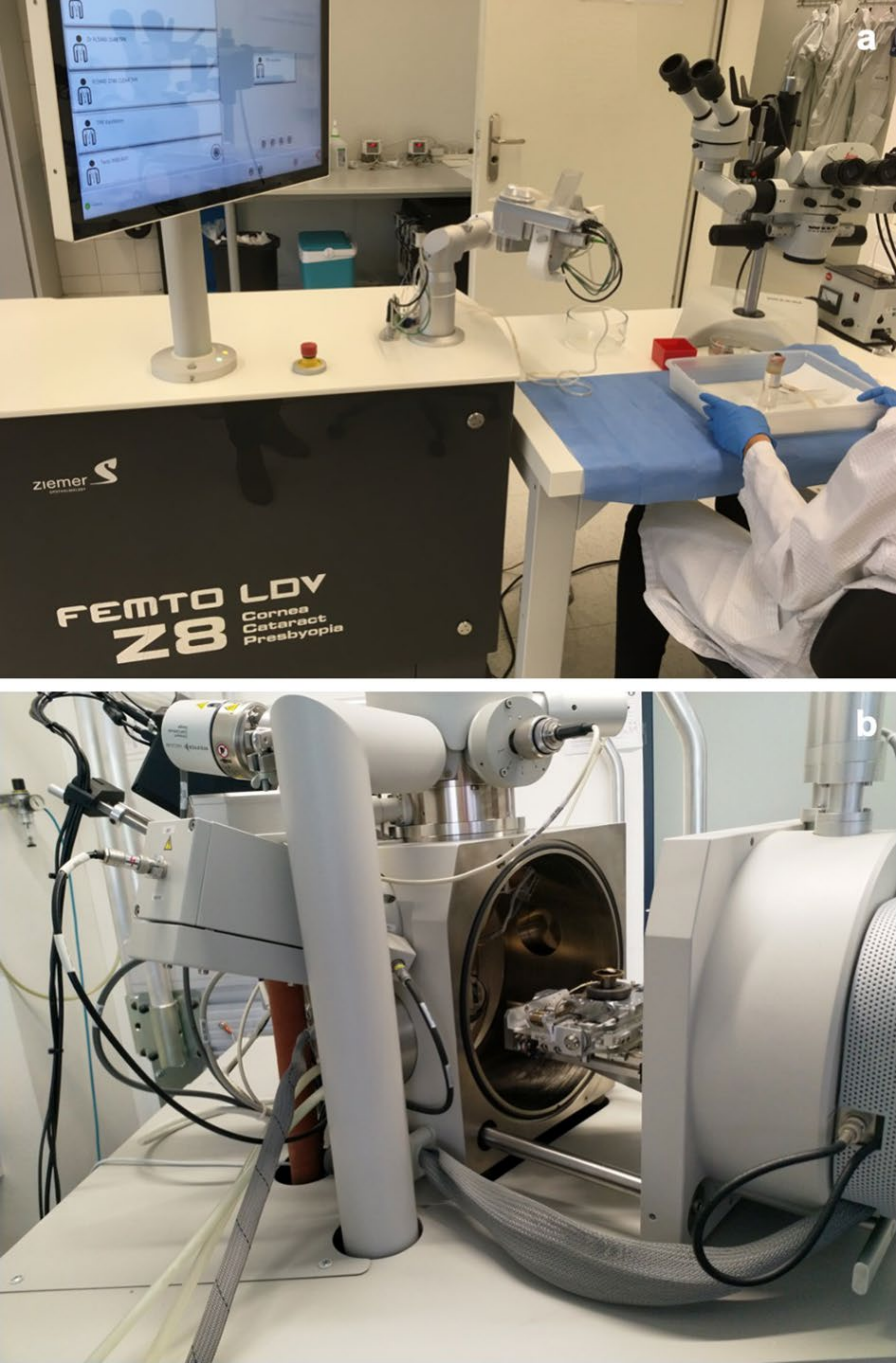
(**a**) Standardised lab set up for *ex-vivo* porcine eye surgeries. (Permission obtained from Ziemer AG, Switzerland for displaying the brand logo). (**b**) Porcine cornea mounting on the Scanning Electron Microscopy.

All surgeries were conducted by the same operator in a standardised setup at Ziemer facilities in Port, Switzerland (Fig. 1a) using FEMTO LDV Z8. This FEMTO LDV Z8 has a wavelength of 1020-1060 nm, a pulse duration of 200-350 fs, and a laser pulse repetition of > 5000 kHz. It has a flat contact glass on docking system with intraoperative OCT, automatic pupil detection and pupil centre offset facility. Preoperatively, the femtosecond laser allowed re-centring of the treatment area if needed. The two 3.0 mm wide incisions were made at 35-degree and 145-degree with an entrance angle of 90 degrees. These incisions allowed the posterior and anterior surfaces of the lenticule to be delineated directly. In all cases, the lenticule diameter was set at 6.6 mm. The vacuum applied to the eye was the same as that used in LASIK flaps (700 bar) during the lenticule cutting stage.

A total of 24 porcine eyes were used in this study.

- Group 1: 6 eyes cut with optimal laser power settings of 32% with a posterior curvature equivalent to a sphere correction of -2D
- Group 2: 6 eyes cut with high laser power settings of 64% with posterior curvature equivalent to a sphere correction of -2D
- Group 3: 6 eyes cut with optimal laser power settings of 32% with posterior curvature equivalent to a sphere correction of -5D
- Group 4: 6 eyes cut with high laser power settings of 64% with posterior curvature equivalent to a sphere correction of -5D

**Figure 2:**
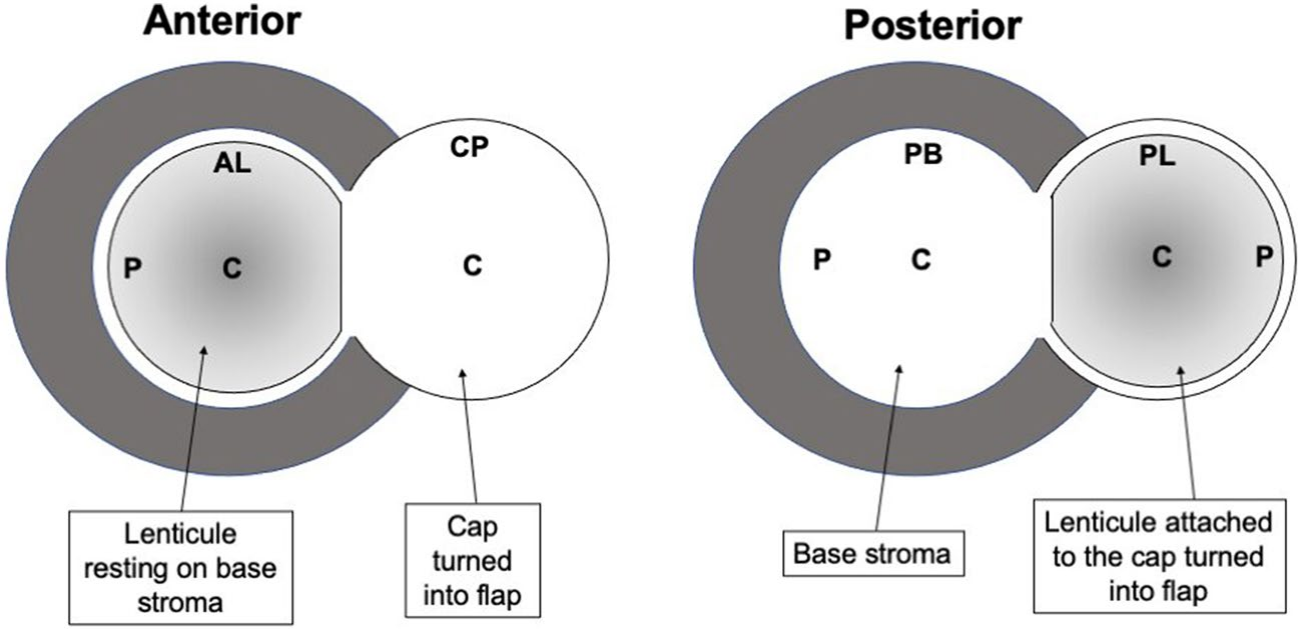
Figure showing areas of scanning when the lenticule was attached to the stromal bed or the cap after the cap was dissected to be lifted like a flap. AL-P = anterior lenticule surface periphery; AL-C = anterior lenticule surface centre; CP-C = Centre of cap’s posterior surface; PL-P = posterior lenticule surface periphery; PB-P = posterior stroma base periphery; PL-C = posterior lenticule surface centre; PB-C = posterior stroma base centre.

After laser application, in the first three eyes in each group, the anterior edge of the lenticule was delineated using a small, pointed spatula (Duckworth & Kent Ltd.) through the first incision. The cap was then dissected like a LASIK flap with Vanna’s scissors and flipped over, leaving the lenticule on the stromal bed to allow SEM scanning on the anterior surface of the lenticule and posterior surface of the dissected cap. A total of three eyes were used for anterior lenticule (AL) scans when the lenticule was resting on the stromal bed (AL-P & AL-C in Fig. 2) and the cap’s posterior surface (CP-C in Fig. 2)(AL = anterior lenticule; P = periphery; C = centre; CP = cap’s posterior surface). Therefore nine images were obtained from 3 eyes. In the remainder of the three eyes in each group, the posterior plane was delineated through the incision, guiding to the posterior plane using a small, pointed spatula (Duckworth & Kent Ltd.). The cap and the attached lenticule were dissected like a LASIK flap and flipped over, leaving the lenticule attached to the flap of the cap to allow SEM scanning of the posterior surface of the lenticule and posterior corneal stromal bed.

Similarly, three eyes were used for imaging the posterior lenticule (PL-C and PL-P in Fig. 2) and posterior base of the corneal stroma (PB-C and PB-P in Fig. 2)(where PL = posterior lenticule; C = centre, P = periphery; PL = posterior lenticule). Therefore, a total of 12 images were obtained from 3 eyes. As six eyes were used in each of the four groups, overall, 24 eyes were used for the entire study.

Immediately after the surgeries in Port, Switzerland, the porcine corneae were transferred to solution 1 [Sörensen-buffer solution + 2% Glutaraldehyde) for up to 4 h before moving to solution 2 (2% Paraformaldehyde + 2.5% Glutaraldehyde 0.1 M Sörensen-buffer solution) to enable the tissue to be stored without affecting it’s morphology for long term storage. The tissue was transported to Laser *Zentrum Hannover* at 4 °C on the same day. The samples were analysed at *Laser Zentrum Hannover* (Hannover, Germany) for scanning electron microscopy (SEM) within a week. The SEM (Quanta 400 FEG, FEI) operated at room temperature (Fig. 1b). The standard air-conditioning at about 22 °C was noted during the procession of all samples in this study. Samples were sputtered with gold and stored at room temperature. Each group and site have three eyes for X400 and X800 magnification images. Scanning was repeated until the most precise images were obtained. The mean processing time for each sample was 20 min after the porcine eyes were docked in the SEM chamber. The resultant image, which included a 200 µm scale marker at X400 magnification and a 100 µm scale marker at X800 magnification, was digitised at a resolution of 2048 dpi X 1760 dpi and saved as an uncompressed image in tiff (tag image file format). This process was standardised for all samples.

The surface morphology of the corneal lenticules was evaluated using a modified scoring system based on the previously published scoring system^9,20,21^ (Table 1). Four selective criteria were used to transform qualitative to quantitative information. The score evaluated the extent of surface irregularities and the position of the irregular area in addition to surface relief. As the criteria are subjective, four independent clinicians graded the findings. We employed untrained clinicians who were not involved in dissection or scanning to grade the images to avoid bias. They were asked to grade the images subjectively based on the scoring system provided.

**Table 1:** Modified scoring system for the quality of the surface.

	Criterion (Original Magnification)	Appearance	Scores
A	Surface relief (X400)	Very smooth	4
		Smooth	3
		Rough	2
		Very rough	1
B	Regularity of surface structure (X800)	Completely regular	4
		Regular	3
		Partially regular	2
		Not regular	1
C	Portion of surface irregular (X800)	Less than 10% of cut surface	4
		11% to 25% of cut surface	3
		26% to 50% of cut surface	2
		More than 50% of cut surface	1
D	Size of the irregular area (X800)	No irregularities	4
		Peripheral only	3
		Large region	2
		All over	1

Data were entered into an Excel software spreadsheet (Microsoft Corp., USA). All further evaluations were performed using standard software (Excel, Microsoft Office 365 for Mac). The mean ± standard deviation (SD) was calculated. T-test was used to compare the mean scores of the image grading between the groups. A P value < 0.05 was considered statistically significant.

## Results

This study included twenty-four fresh porcine eyes (6 in each group). A total of 84 images (21 in each group) were available for analysis. None of the eyes had any surgical complications, and none were discounted for the analysis due to any reason. No holes (dents on the surface) from cavitation bubbles were seen in any images.

**Figure 3:**
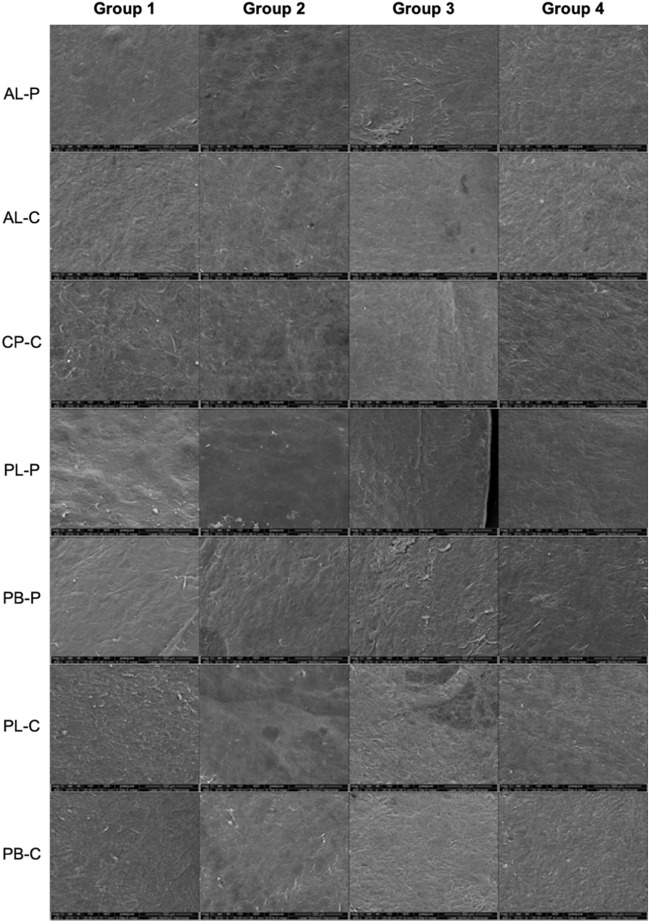
Example SEM pictures at X400 magnification for scans at different locations in group 1 (laser power:32% for − 2.0D treatment), group 2 (laser power:64% for − 2.0D treatment), group 3 (laser power:32% for − 5.0D treatment) and group 4 (laser power:64% for − 5.0D treatment). The pictures show the scans of anterior lenticular surface periphery & centre, anterior cap centre, posterior lenticular periphery & centre and posterior stromal base centre and periphery. (AL-P = anterior lenticule surface periphery; AL-C = anterior lenticule surface centre; CP-C = Centre of cap’s posterior surface; PL-P = posterior lenticule surface periphery; PB-P = posterior stroma base periphery; PL-C = posterior lenticule surface centre; PB-C = posterior stroma base centre.).

**Figure 4:**
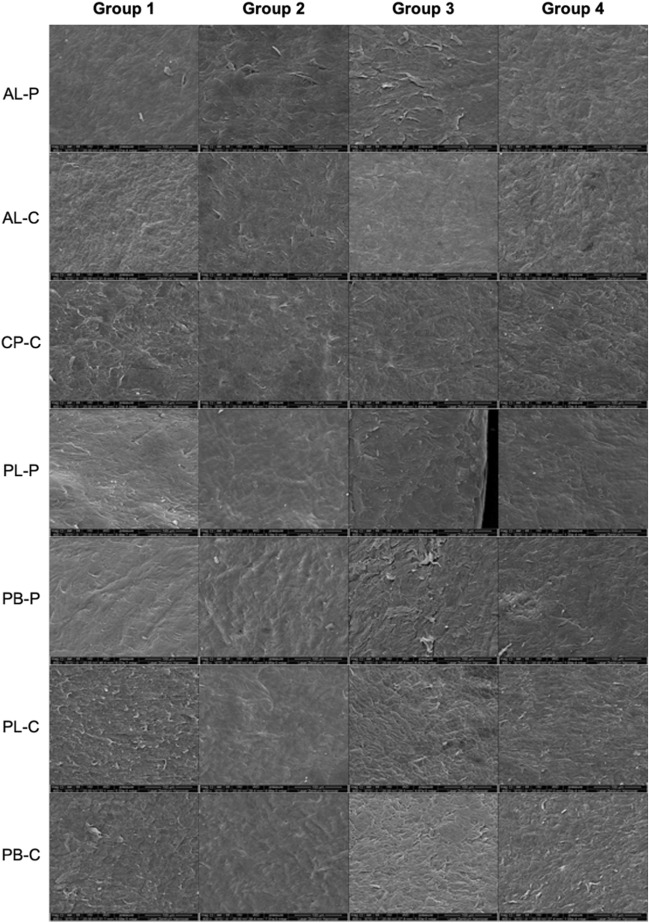
Example SEM pictures at X800 magnification for scans at different locations in group 1 (laser power:32% for − 2.0D treatment), group 2 (laser power:64% for − 2.0D treatment), group 3 (laser power:32% for − 5.0D treatment) and group 4 (laser power:64% for − 5.0D treatment). The pictures show the scans of anterior lenticular surface periphery & centre, anterior cap centre, posterior lenticular periphery & centre and posterior stromal base centre and periphery. (AL-P = anterior lenticule surface periphery; AL-C = anterior lenticule surface centre; CP-C = Centre of cap’s posterior surface; PL-P = posterior lenticule surface periphery; PB-P = posterior stroma base periphery; PL-C = posterior lenticule surface centre; PB-C = posterior stroma base centre.)

Example images obtained for X400 and X800 magnification are shown in Figs. 3–4. The mean scores of 4 independent graders are shown in Table 2. The comparison analysis is given in Table 3.

**Table 2:** Mean scores from all 4 graders in all 4 groups (group 1: 32% laser power for − 2.0D treatment), group 2: 64% laser power for − 2.0D treatment, group 3: 32% laser power for − 5.0D treatment and group 4: 64% laser power for − 5.0D treatment). The anterior lenticular surface periphery & centre, anterior cap centre, posterior lenticular periphery & centre and posterior stromal base centre and periphery scans were analysed. (AL-P = anterior lenticule surface periphery; AL-C = anterior lenticule surface centre; CP-C = centre of cap’s posterior surface; PL-P = posterior lenticule surface periphery; PB-P = posterior stroma base periphery; PL-C = posterior lenticule surface centre; PB-C = posterior stroma base centre).

		A : Surface relief (X400) (mean ± standard deviation)	B : Regularity of surface structure (X800) (mean ± standard deviation)	C : Portions of irregular surface (X800) (mean ± standard deviation)	D : Size of the irregular area (X800) (mean ± standard deviation)
Group 1	**AL-P**	2.89 ± 0.60	2.33 ± 0.50	3.11 ± 0.78	2.89 ± 0.78
	**AL-C**	2.89 ± 0.93	2.67 ± 0.71	3.22 ± 0.97	3.00 ± 0.87
	**CP-C**	2.56 ± 0.73	2.33 ± 0.87	3.00 ± 1.00	2.44 ± 1.01
	**PL-P**	2.33 ± 0.71	1.78 ± 0.67	2.78 ± 1.2	2.33 ± 0.71
	**PB-P**	2.78 ± 0.97	2.44 ± 0.88	2.89 ± 1.17	2.22 ± 0.83
	**PL-C**	2.22 ± 0.44	2.33 ± 0.50	2.33 ± 1.12	2.33 ± 0.87
	**PB-C**	2.22 ± 0.44	2.11 ± 0.78	2.67 ± 1.12	2.11 ± 0.78
Group 2	**AL-P**	2.67 ± 0.50	1.89 ± 0.60	2.44 ± 1.42	2.44 ± 0.53
	**AL-C**	2.67 ± 0.87	2.11 ± 0.78	2.56 ± 1.51	2.89 ± 0.93
	**CP-C**	2.67 ± 0.71	2.11 ± 0.60	2.67 ± 1.12	2.78 ± 0.67
	**PL-P**	2.56 ± 0.73	2.22 ± 0.97	3.00 ± 0.87	2.89 ± 0.60
	**PB-P**	2.56 ± 0.73	1.89 ± 0.60	2.33 ± 1.22	2.22 ± 0.97
	**PL-C**	2.44 ± 0.88	2.11 ± 0.78	3.22 ± 0.97	2.56 ± 0.53
	**PB-C**	2.44 ± 0.73	2.22 ± 0.67	2.89 ± 1.05	2.78 ± 0.83
Group 3	**AL-P**	2.11 ± 0.93	2.00 ± 1.00	2.00 ± 1.32	1.89 ± 1.05
	**AL-C**	2.56 ± 1.01	2.00 ± 0.71	2.22 ± 1.2	2.11 ± 0.78
	**CP-C**	2.44 ± 0.73	2.33 ± 1.12	2.44 ± 1.51	2.22 ± 1.09
	**PL-P**	1.89 ± 0.78	1.67 ± 0.71	2.22 ± 1.39	1.89 ± 0.93
	**PB-P**	2.22 ± 0.83	2.11 ± 0.93	2.44 ± 1.51	2.11 ± 0.93
	**PL-C**	2.22 ± 0.44	2.56 ± 0.73	3.00 ± 1.32	2.78 ± 0.97
	**PB-C**	2.56 ± 0.53	2.33 ± 0.50	3.00 ± 1.32	2.78 ± 1.09
Group 4	**AL-P**	2.33 ± 1.00	2.00 ± 0.87	2.44 ± 1.24	2.33 ± 0.87
	**AL-C**	2.67 ± 0.71	2.56 ± 0.88	3.00 ± 1.32	2.89 ± 1.17
	**CP-C**	2.67 ± 1.00	2.67 ± 1.00	2.78 ± 1.30	2.56 ± 1.13
	**PL-P**	2.89 ± 0.60	2.33 ± 0.71	2.89 ± 1.27	2.56 ± 0.73
	**PB-P**	2.33 ± 0.50	1.56 ± 0.73	2.44 ± 1.24	2.22 ± 0.97
	**PL-C**	2.67 ± 0.71	2.33 ± 0.71	2.78 ± 1.30	2.33 ± 0.87
	**PB-C**	2.56 ± 0.53	2.33 ± 0.5	3 ± 1.12	2.67 ± 0.71

**Table 3:** P values comparing various groups. Group 1: 32% laser power for − 2.0D treatment), group 2: 64% laser power for − 2.0D treatment, group 3: 32% laser power for − 5.0D treatment and group 4: 64% laser power for − 5.0D treatment). The anterior lenticular surface periphery & centre, anterior cap centre, posterior lenticular periphery & centre and posterior stromal base centre and periphery scans were analysed. (AL-P = anterior lenticule surface periphery; AL-C = anterior lenticule surface centre; CP-C = centre of cap’s posterior surface; PL-P = posterior lenticule surface periphery; PB-P = posterior stroma base periphery; PL-C = posterior lenticule surface centre; PB-C = posterior stroma base centre.)

		A : Surface relief (X400)	B : Regularity of surface structure (X800)	C : Portions of irregular surface (X800)	D : Size of the irregular area (X800)
Group 1 vs 3	AL-P	0.05	0.38	0.05	0.04*
	AL-C	0.48	0.06	0.07	0.04*
	CP-C	0.75	1.00	0.37	0.66
	PL-P	0.22	0.74	0.38	0.27
	PB-P	0.21	0.45	0.49	0.79
	PL-C	1.00	0.46	0.27	0.32
	PB-C	0.16	0.48	0.57	0.16
Group 2 vs 4	AL-P	0.38	0.76	1.00	0.75
	AL-C	1.00	0.27	0.52	1.00
	CP-C	1.00	0.17	0.85	0.62
	PL-P	0.30	0.79	0.83	0.30
	PB-P	0.46	0.30	0.85	1.00
	PL-C	0.56	0.54	0.42	0.52
	PB-C	0.72	0.69	0.83	0.76
Group 1 vs 2	AL-P	0.45	0.10	0.14	0.04*
	AL-C	0.17	0.05	0.14	0.76
	CP-C	0.68	0.51	0.44	0.35
	PL-P	0.51	0.27	0.59	0.10
	PB-P	0.59	0.21	0.38	1.00
	PL-C	0.51	0.35	0.04*	0.35
	PB-C	0.17	0.68	0.51	0.05
Group 3 vs 4	AL-P	0.63	1.00	0.47	0.34
	AL-C	0.79	0.16	0.21	0.12
	CP-C	0.60	0.51	0.62	0.53
	PL-P	0.01*	0.06	0.30	0.11
	PB-P	0.74	0.18	1.00	0.81
	PL-C	0.13	0.52	0.72	0.32
	PB-C	1.00	1.00	1.00	0.80

*statistically significant.

### Group 1 (− 2.0D with 32% laser power) vs. Group 3 (− 5.0D with 32% laser power)

Group 1 had significantly less ‘size of the irregular area (× 800)’ for AL-P and AL-C (Tables 2 and 3), suggesting more irregularities only towards the periphery with − 2.0D with 32% power compared to irregularities all over in − 5.0D with 32% laser power for AL-P and AL-C regions.

### Group 2 (− 2.0D with 64% laser power) vs. Group 4 (− 5.0D with 64% laser power)

We found no significant difference in any parameter for any scan locations (Tables 2 and 3).

### Group 1 (− 2.0D with 32% laser power) vs. Group 2 (− 2.0D with 64% laser power)

We found significantly less ‘size of the irregular area’ for AL-P with group 1, suggesting that the − 2.0D with 32% laser power created irregularities mainly in the periphery compared to larger regions with − 2.0D with 64% laser power. ‘Proportion of irregular areas’ were significantly different with group 2 compared to group 1 for PL-C (Tables 2 and 3), suggesting that the majority of the samples showed an 11%-25% proportion of irregular surface with − 2.0D with 32% laser power compared to 26%-50% with − 2.0D with 64% laser power.

### Group 3 (− 5.0D with 32% laser power) vs. Group 4 (− 5.0D with 64% laser power)

We found significantly less ‘surface relief’ at X400 for PL-P with group 3 compared to group 4 (Tables 2 and 3), suggesting that the posterior lenticular periphery was more on the rougher side with − 5.0D with 64% laser power and on the smoother side with 32% laser power.

## Discussion

In this *ex-vivo* study on porcine eyes, comparing − 2.0D and − 5.0D for the lower laser power of 32%, we found a smooth anterior surface with irregularities observed only towards the periphery with the − 2.0D group. Whereas with − 5.0D, the irregularities were observed all over the surface of the lenticule. For higher laser power of 64%, we did not find any difference between − 2.0D and − 5.0D. When comparing lower (32%) and higher (64%) laser power, for − 2.0D, for the anterior lenticule, lower laser power created irregularities mainly in the peripheral region compared to all over for high laser power. For posterior lenticule, a relatively lesser proportion of irregular area was found with lower laser power. For the same comparison with − 5.0D, the posterior lenticular periphery for − 5.0D was smoother with lower laser power (32%) than higher laser power (64%).

The surface quality of the femtosecond laser corneal lenticule has been assessed in some studies. The earlier studies used a VisuMax laser with a 200-kHz repetition rate, 180 nJ pulse energy, and 3 × 3 μm spot separation in the porcine eye model^9^. Heichel et al.^9^ found that although the surface quality was predictable, it had poor quality than the lenticules cut with a mechanical microkeratome. The quality was poor as they showed surface irregularities (i.e. roughness due to tissue bridges, cavitation bubbles, and grooves). Kunert et al*.*^7^ used a 200-kHz VisuMax system with a 3 × 3 μm spot separation and three pulse energies (150, 180, and 195 nJ) to study the surface quality of lenticules extracted from patients. Using a standardised subjective scale to grade the stromal bed, lenticule appearance, and ease of dissection, they concluded that best quality resections were generated with the lowest pulse energy used (150 nJ), suggesting that lower energy levels produced smoother surfaces^7^. Previous clinical studies have also shown that lower energy levels for lenticule extraction produce superior visual results^22,23^. In our study, rather than increasing the laser power in smaller steps like Kunert et al.^7^, we doubled the laser power from 32 to 64% and although we saw some differences in the surface quality between the laser power settings (Tables 2 and 3) this was comparable with the photographs from Kunert et al.’s^7^ study. We compared lower and higher energy to analyse the surface quality, as the FEMTO LDV Z8 allows the surgeons to adjust the energy levels in individual cases. The VisuMax femtosecond laser has a 500-kHz repetition rate, 130 nJ pulse energy, and 3 × 3 μm spot separation. A study by Ang et al. used cadaver eyes^2^. Whereas, the FEMTO LDV Z8 used in our study has a > 5000-kHz repetition rate, < 100 nJ pulse energy and < 2 µm spot size with overlapping spots to enable even closer spot separation. The previous studies show that pulse energy and laser frequency are essential variables for optimising surface quality when using femtosecond laser for corneal refractive surgery. During photodisruption of the stroma, tiny cavitation bubbles form at the interface. Decreasing the pulse energy and increasing the laser frequency can reduce this bubble occurrence by enabling a more homogeneous cut^24,25^. Spot separation is another critical parameter as the femtosecond laser works through photodisruption of the stroma; many collagen fibres remain attached after sectioning^26^. Some studies on the morphology of LASIK flaps found that a smaller spot separation caused less trauma to the flap and the stroma during flat lifting^27^. In previous studies of lenticule surface quality, a 3 × 3 μm spot separation was used^2,7,9^. Closer spot placement (2.5 × 2.5 μm) will allow for more efficient and easier lenticule extraction as few collagen fibres are left intact^10^. Therefore, reducing laser spot separation is vital to reducing surface roughness^10^.

There is some evidence of regression following SMILE in high myopia, indicative of some difference in the ablation patterns between lower and higher diopters of myopia^28^. We also know that the most typical range of myopia for laser correction is around 4-5D. Taking the potential effect of the magnitude of correction and the contribution of the laser energy, we designed the study to look at -2D and -5D and at low and high energy settings. With the low-energy FEMTO LDV Z6, Riau et al.^29^ documented faster wound healing and showed less flap adhesion strength with FEMTO LDV Z6 than the VisuMax. Although no previous studies focus on the interface adhesion of lenticules during SMILE procedures, Wang et al*.*^16^ found that the FEMTO LDV Z8 lenticule separation was much easier than the VisuMax, which was postulated to be related to the energy-frequency differences of femtosecond lasers during intrastromal incisions^16^. It is impossible to directly compare our SEM images with Wang et al.^16^ due to the differences in magnification, but they appear similar. Several parameters of the femtosecond laser, such as pulse energy, frequency, pulse duration, spot separation, and pulse pattern, will affect the cut morphology and surface quality. The FEMTO LDV Z8 has a higher frequency with low-energy pulses, which reduces the cavitation bubble formation compared to VisuMax. Hence, the FEMTO LDV Z8 produces a complete and smooth stromal interface for easy lenticule separation by reducing the spot separation.

The advantages of lenticule extraction compared to FS-LASIK have also been shown microscopically. Luft et al*.*^30^ found identical and minimal keratocyte activation, cell death, and inflammation after comparing lenticule extraction and FS-LASIK in human cadaver donors corneae. Reactive stromal fibrosis was more following FS-LASIK, which was postulated due to the two different laser wavelengths resulting in differences in the applied laser energy in FS-LASIK^30^. However, Luft et al.^30^ also found a more regular appearance of the stromal bed structurally with FS-LASIK due to the smoothing effect of the excimer laser compared with the SMILE lenticule bed^30^. Higher surgical refractive corrections might accentuate the difference in the wound healing pattern between SMILE and FS-LASIK as per Luft et al.^30^ This is because the energy delivered in the SMILE procedure is constant and independent of the refractive error corrected.

Some of the roughness of the surface quality noted in all previous studies using SEM^2,7,9^ could also be due to the tissue preparation techniques (critical drying point and gold coating) prior to imaging. Previous studies noted shrinkage of samples of up to 60%, leading to contraction and cracking^10,31^. Like previous studies, we used eSEM to image various lenticule surfaces. The eSEM adapts the traditional SEM system to scan hydrated, uncoated biological samples, thereby eliminating artefacts due solely to tissue preparation^10,31^. In our study, we sputtered the sample with gold and stored it at room temperature before the eSEM, and therefore the images were free of artefacts. This gold sputtering also reduced the malleability of our samples, allowing them to be placed entirely flat for imaging without any folds or wrinkles. The presence of water on the surface during eSEM imaging protects the sample from dehydration, but it may reduce contrast and obscure more minor surface details due to vapour release. However, coating gold before eSEM reduces this effect^31,32^. It is already established that the eSEM is a very reliable technique for imaging delicate biological samples. However, lower magnification and a shorter time for imaging will minimise tissue damage^32^.

The strength of our study is that it is the first study employing a new methodology to analyse the surface quality after dissecting the cap off the lenticule and dissecting the cap with the lenticule from the stromal bed. As the surface quality of the stromal bed and cap is more important to study, we employed a tissue dissection technique (Fig. 1) to give lesser eSEM artefacts from tissue trauma during the dissection than the clinical protocol where the lenticule is removed after dissection. We did not analyse the anterior cap’s periphery (CP-P) due to the above reason. There are a few limitations to our study. First, CLEAR was performed on porcine eyes rather than human cadaver eyes. Because this study was conducted during the COVID-19 pandemic procuring human cadaver eyes was complex. Therefore porcine cornea, which has been widely used in previous studies, was chosen as a close model for human cornea. The porcine eyes study may not be directly comparable to the human cadaveric eye and/or live subjects. Therefore in this study we did not compare the surgical technicalities regarding the ease of lenticule extraction and dissection between the groups. Second, we did not analyse cap thickness in this study; however, it was set as standard at 120 µm for all cases during the procedure. The conventional setting of cap thickness during SMILE is 120 to 140 µm^33,34^. Although a thicker cap thickness setting in SMILE was reported to have a more negligible effect on the corneal biomechanics^25^, deeper stromal ablations might decrease laser efficacy^25^. Moreover, it is also known that thicker caps are associated with more tissue bridges compared to thinner caps^8,21^. Third, we did not assess the surface quality for high myopia over -6D, astigmatism and hyperopia. We chose to analyse up to -5D only based on the average preoperative spherical equivalent published in a meta-analysis and systematic review by Wu et al*.y*^35^. Fourth, this experiment was performed on an uncommercially available module. FEMTO LDV Z8 CLEAR has not been accessible in clinical practice to validate our findings in clinical settings. Fifth, the energy parameters between different lasers for lenticule extractions cannot be directly compared. FEMTO LDV Z8 uses percentage because the technology is based on overlapping pulses, and the energy depends on several parameters. In contrast, other platforms express the energy in the nanojoules unit. Finally, the sample size of the eyes may be small. Still, we have analysed multiple areas from each dissection and decided to use four blinded graders who independently scored the images. We also ensured that these graders were clinicians, and grading was performed on the overall appearance of SEM pictures only. Moreover, the grading system is subjective and may have subjective biases like the previous studies. To reduce the impact of these biases, we recruited four blinded graders for independent scoring.

In our *ex-vivo* study on porcine eyes, for − 2.0D and − 5.0D, the surface, in general, was more on the smoother side with lower laser power compared to higher laser power. Anterior lenticular changes were also lesser with lower laser power (32%) than higher laser power (64%) for − 2.0D myopic correction, which may be attributable to the low energy and overlapping spot density combination of the FEMTO LDV Z8 rather than just lower energy settings.
